# Perceived Influence of Incentives on COVID-19 Vaccination Decision-making and Trust

**DOI:** 10.1001/jamanetworkopen.2023.13436

**Published:** 2023-05-19

**Authors:** Laura J. Faherty, Gerald P. Hunter, Pierrce Holmes, Jeanne S. Ringel

**Affiliations:** 1 RAND Corporation, Boston, Massachusetts; 2 Department of Pediatrics, Maine Medical Center, Portland; 3 Department of Pediatrics, Tufts University School of Medicine, Boston, Massachusetts; 4 RAND Corporation, Pittsburgh, Pennsylvania; 5 RAND Corporation, Santa Monica, California

## Abstract

This survey study examines the prevalence of incentive receipt for COVID-19 vaccination and the association of various sociodemographic characteristics with perspectives on incentives’ influence on trust in the COVID-19 vaccine.

## Introduction

Evidence remains sparse and mixed regarding the use of incentives to promote COVID-19 vaccination.^1,2,3,4^ This survey study examined the prevalence of incentive receipt for COVID-19 vaccination overall and by sociodemographic group, the association of incentives with COVID-19 vaccine uptake, and the association of sociodemographic characteristics with perceived influence of incentives on trust in the COVID-19 vaccine.

## Methods

This survey study was approved by the RAND Corporation institutional review board and participants consented to participate through a survey question. We followed the American Association for Public Opinion Research (AAPOR) reporting guideline. An online survey was conducted between June 15 and June 23, 2022, using the RAND American Life Panel, yielding nationally representative estimates from a probability-based sample of the US adult population. Additional details are provided in the eAppendix and eTable in Supplement 1. Self-reported race and ethnicity were categorized as Asian or Pacific Islander, Black or African American, Hispanic, White, or other race and ethnicity (including American Indian or Alaska Native or other race not listed). Race and ethnicity were included to allow for analyses comparing the outcomes of interest across different racial and ethnic groups. Descriptive statistics were calculated, and frequencies and proportions were cross-tabulated. We used χ^2^ tests to compare sociodemographic characteristics and vaccination status, the proportion of respondents who received an incentive if vaccinated, and the extent to which respondents perceived incentives to influence their trust in the COVID-19 vaccine. Analyses were conducted using Stata MP, version 17.0 (StataCorp, LLC). Statistical significance was a 2-sided *P* < .05. Data were analyzed from July to November 2022.

## Results

Table 1 summarizes the sociodemographic characteristics of the 2356 respondents (1332 [52.%] women; 950 respondents [22%] aged ≥65 years) who completed the survey and lists the proportion of respondents who received an incentive, if vaccinated. Of 2356 respondents, 67 (4%) were Asian or Pacific Islander, 208 (12%) were Black or African American, 295 (18%) were Hispanic and 1729 (62%) were White. A total of 552 respondents (23%) reported residing in a rural area or a small town; 517 (26%) reported an income of less than $35 000 per year, and 310 (38%) reported having earned a high school diploma or less. A total of 2042 individuals (87% unweighted, 83% weighted) had received at least 1 dose of the COVID-19 vaccine.

**Table 1.** Sociodemographic Characteristics of the Sample, Overall and by Vaccination Status and Receipt of Incentive^a^

Characteristic	Total respondents, No. (weighted %) [95% CI] (N = 2356)	Vaccination status, No. (weighted %) [95% CI]		*P* value	Vaccinated respondents who received an incentive, No. (weighted %) [95% CI] (n=2042)	*P* value
		Vaccinated (n = 2042)	Unvaccinated (n = 314)			
Gender						
Women	1332 (52) [48-56]	1131 (49) [45-54]	201 (63) [54-70]	.005	80 (9) [6-12]	.72
Men	1024 (48) [44-52]	911 (51) [46-55]	113 (37) [30-46]		56 (8) [5-13]	
Age, y						
18-29	83 (17) [12-22]	71 (17) [13-24]	12 (13) [7-21]	.03	11 (11) [5-21]	.004
30-39	255 (19) [17-23]	209 (18) [15-22]	46 (25) [18-34]		28 (16) [10-24]	
40-49	321 (17) [14-19]	253 (16) [13-19]	68 (21) [15-28]		24 (10) [5-20]	
50-64	747 (25) [23-28]	641 (25) [22-28]	106 (29) [22-36]		45 (6) [4-9]	
≥65	950 (22) [20-25]	868 (24) [21-27]	82 (13) [10-18]		28 (3) [2-5]	
Race and ethnicity						
Asian or Pacific Islander	67 (4) [3-6]	64 (5) [3-7]	3 (1) [0-3]		5 (6) [2-19]	.002
Black or African American	208 (12) [10-15]	177 (12) [9-15]	31 (12) [8-18]		24 (19) [11-30]	
Hispanic	295 (18) [15-30]	247 (18) [15-21]	48 (19) [13-28]		24 (13) [7-23]	
White	1729 (62) [59-66]	1505 (62) [57-66]	224 (66) [57-74]		81 (6) [4-8]	
Other^b^	57 (4) [2-6]	49 (4) [3-7]	8 (2) [1-5]		2 (2) [0-9]	
Urbanicity						
City or urban area	1807 (77) [74-80]	1591 (79) [76-82]	210 (70) [62-76]	.01	101 (8) [6-11]	.32
Rural or small town	552 (23) [20-26]	450 (21) [18-24]	102 (30) [24-38]		35 (10) [6-17]	
Family income, $						
<35 000	517 (26) [23-29]	406 (21) [18-25]	111 (48) [40-56]	<.001	35 (13) [8-20]	.001
35 000-59 999	486 (19) [16-23]	407 (19) [16-23]	79 (20) [15-27]		34 (8) [5-12]	
60 000-99 999	585 (24) [20-28]	518 (25) [20-30]	67 (18) [13-25]		31 (10) [5-18]	
≥100 000	764 (31) [28-35]	709 (35) [31-39]	55 (14) [10-19.6]		36 (6) [4-9]	
Education level						
≤High school diploma	310 (38) [34-43]	228 (35) [30-40]	82 (55) [48-63]	<.001	13 (7) [4-14]	.09
Some college or associate’s degree	738 (27) [24-30]	609 (27) [23-30]	129 (28) [22-34]		53 (11) [8-17]	
Bachelor’s degree	675 (19) [17-21]	602 (20) [18-23]	73 (13) [9-18]		37 (8) [6-13]	
≥Master’s degree	633 (16) [14-18]	603 (19) [16-21]	30 (4) [3-6]		33 (8) [5-12]	

^a^ All variables had complete data except US region (1 missing response); urbanicity (3 missing responses); income (4 missing responses); and general health (9 missing responses).

^b^ Other race or ethnicity refers to participants who self-identified as a race or ethnicity not listed, or who self-identified as American Indian or Alaska Native.

Of the 2042 vaccinated respondents, 136 respondents (9%) reported receiving an incentive, commonly employment-related (eg, paid time off) or a gift card. Of respondents who received an incentive, 101 (64%) reported that an incentive made no difference in their decision to get vaccinated. The proportion of respondents who received an incentive varied by age group and race and ethnicity (5 Asian or Pacific Islander individuals [6%], 24 Black or African American individuals [19%], 24 Hispanic individuals [13%], and 81 White individuals [6%]) (Table 1). Respondents with the highest income (>$100 000 per year) were least likely to have received an incentive.

Race and ethnicity, urbanicity, income, and education were significantly associated with perceptions of incentives’ influence on trust in vaccination (Table 2). Approximately 20% of Asian or Pacific Islander, Black and Hispanic respondents reported that incentives increased their trust in the COVID-19 vaccine, compared to 4% of White respondents (47 respondents). Rural respondents were significantly more likely than urban respondents to report that incentives eroded their trust in vaccination (166 respondents [34%] vs 351 respondents [22%]; *P* < .001). As income or educational attainment increased, an increasing proportion of respondents indicated that the incentive did not have any influence on their trust.

**Table 2.** Survey Respondents’ Perceptions of the Influence of Incentives on Trust in the COVID-19 Vaccine^a^

Characteristic	Perceived influence of incentive on trust in COVID-19 vaccine, No. (Weighted %) [95% CI] (N=2361)			*P* value
	More trust	No change	Less trust	
Overall	123 (9) [7-12]	1719 (66) [62-70]	519 (25) [22-28]	
Gender				
Women	69 (9) [6.4-12]	979 (65) [61-69]	288 (26) [23-30]	.73
Men	54 (10) [6-14]	740 (67) [61-73]	231 (23) [19-29]	
Age, y				
18-29	9 (11) [5-23]	57 (73) [59-83]	17 (16) [9-27]	.070
30-39	17 (12) [7-18]	176 (59) [50-67]	62 (30) [22-38]	
40-49	18 (9) [4-18]	211 (59) [50-68]	92 (32) [24-40]	
50-64	41 (8) [5-12]	535 (65) [59-70]	176 (27) [23-33]	
≥65	38 (7) [4-11]	740 (74) [70-78]	172 (19) [16-23	
Race and ethnicity				
Asian or Pacific Islander	8 (21) [7-47]	47 (60) [39-78]	12 (20) [9-39]	
Black or African American	25 (21) [13-33]	145 (59) [48-70]	38 (20) [13-29]	
Hispanic	42 (17) [12-25]	179 (55) [46-64]	74 (27) [20-36]	
White	47 (4) [2-7]	1302 (70) [65-74]	384 (26) [23-30]	
Other^b^	1 (3) [0-18]	46 (85) [70-93]	11 (12) [5-26]	
Urbanicity				
City or urban area	108 (11) [8-14]	1346 (67) [63-71]	351 (22) [19-26]	<.001
Rural or small town	15 (4) [2-8]	372 (62) [55-68]	166 (34) [28-41]	
Family income, $				
<35 000	53 (15) [11-22]	339 (53) [46-59]	127 (32) [26-39]	<.001
35 000-59 999	26 (12) [6-20]	333 (63) [55-71]	129 (25) [19-32]	
60 000-99 999	23 (6) [3-12]	443 (71) [63-78]	120 (22) [16-30]	
≥100 000	21 (5) [2-9]	601 (75) [70-80]	142 (21) [17-25]	
Education level				
≤High school diploma	38 (14) [10-21]	178 (56) [48-64]	95 (29) [23-37]	<.001
Some college or associate’s degree	44 (8) [5-13]	494 (67) [62-72]	202 (25) [21-30]	
Bachelor’s degree	22 (5) [3-9]	512 (71) [65-76]	141 (24) [20-30]	
≥Master’s degree	19 (4) [2-8]	535 (81) [76-86]	81 (15) [11-20]	

^a^ Results are reported regardless of vaccination status due to small cell sizes in the unvaccinated sample.

^b^ Other race or ethnicity refers to participants who self-identified as a race or ethnicity not listed, or who self-identified as American Indian or Alaska Native.

## Discussion

This survey study found that although there has been substantial policy attention around incentivizing COVID-19 vaccination, fewer than 1 in 10 vaccinated individuals in a nationally representative sample of US adults reported receiving an incentive. Most vaccinated respondents reported that an incentive did not make a difference in their decision-making to get the COVID-19 vaccine. Incentive receipt was more common among individuals from minoritized racial and ethnic groups such as Asian or Pacific Islander, Black, or Hispanic individuals. These findings could indicate successful targeting of incentives to those who would most benefit from COVID-19 vaccination because of disproportionate morbidity and mortality.^5^ These findings could indicate there was a greater need for incentivization for these individuals or that these individuals were more willing to get vaccinated if offered an incentive.

The finding that perceptions of incentives’ influence on trust in the COVID-19 vaccine varied by sociodemographic characteristics highlights the importance of tailored outreach to promote vaccination^6^ and consideration of whether incentives will have the desired outcome in subpopulations of interest. The strong associations of trust with urbanicity, education, and income may be useful to public health officials seeking to collaborate with communities to codesign effective and sustainable incentive strategies to reach unvaccinated groups.

Study limitations include lack of data on vaccination timing, incentive source, and associations among sociodemographic characteristics. Another limitation of the study is the potential for social desirability and recall bias.

## Notes

Supplement 1. **eAppendix.** Supplemental Methods: DataData Collection and Statistical Analysis **eTable.** Survey Questions and Response Options

Supplement 2.
Data Sharing Statement
