# Supplementary material for: Perceived Influence of Incentives on COVID-19 Vaccination Decision-making and Trust
Source: JAMA Netw Open. 2023 May 19;6(5):e2313436. doi: 10.1001/jamanetworkopen.2023.13436 (PMC10199349; doi:10.1001/jamanetworkopen.2023.13436)
Supplement: Supplement 1. — eAppendix. Supplemental Methods: DataData Collection and Statistical Analysis eTable. Survey Questions and Response Options [file jamanetwopen-e2313436-s001.pdf]

Supplemental Online Content

Faherty LJ, Hunter GP, Holmes P, Ringel JS. Perceived influence of incentives on COVID-19 vaccination decision-making and trust. *JAMA Netw Open*. 2023;6(5):e2313436. doi:10.1001/jamanetworkopen.2023.13436

eAppendix. Supplemental Methods: Data Collection and Statistical Analysis
eTable. Survey Questions and Response Options

This supplemental material has been provided by the authors to give readers additional information about their work.

© 2023 Faherty LJ et al. *JAMA Network Open.*

eAppendix. Supplemental Methods

Data Collection

We conducted an online survey using the RAND American Life Panel, which yields nationally representative estimates from a probability-based sample of the US adult population aged 18 years or older. The [*blinded for review*] Institutional Review Board approved the survey with a waiver of informed consent. We followed the American Association for Public Opinion Research (AAPOR) reporting guideline.

The American Life Panel recruited an initial wave of participants in 2002 and is refreshed on an ongoing basis to create a total current panel size of over 6,000 participants. As described previously, participants are recruited using a variety of methods to ensure population-representativeness (e.g., sampling in zip codes with high percentages of racial minority groups), are provided internet-enabled devices, and are paid for completing the survey. (For more information, please see Pollard M, Baird M. *The RAND American Life Panel: Technical description.* Santa Monica, CA: RAND Corporation;2017. RR1651).

The data for the present study were collected through the American Life Panel Omnibus Survey Wave 12, which was fielded between June 15 and 23, 2022. This survey coincided with a period during which two Omicron subvariants of the novel coronavirus (BA.4 and BA.5) were becoming dominant in the United States. RAND uses a raking procedure to weight the survey to national demographic distributions from the U.S. Current Population Survey.

We asked a series of questions (see **Table S1** in the Supplement) about the participants' vaccination status, which we dichotomized into those who had received at

© 2023 Faherty LJ et al. *JAMA Network Open.*

least one dose of a COVID-19 vaccine and those who had not and whether those who were vaccinated received any type of incentive (response options: yes, no, unsure). If the respondent indicated that they had received an “incentive,” we asked them to write-in a brief description of the incentive they received in a free-text box. We then sorted the free-text responses into categories using an inductive approach. We also asked them to indicate on a Likert scale to what extent the incentive influenced their vaccination decision. For those who had not received a dose of a COVID-19 vaccine, we asked them if they were to receive a $50 gift card, to what extent this incentive would influence their likelihood of getting vaccinated. Finally, we asked all respondents to indicate on a five-point Likert scale ranging from “a lot more” to “a lot less,” the extent to which incentives influence their trust in COVID-19 vaccination. We trichotomized Likert scale response options for ease of interpretation.

Statistical Analysis

Using the American Life Panel response weights, we produced nationally representative estimates of answers to our survey questions. We calculated descriptive statistics, including frequencies and proportions (with confidence intervals when small cell sizes precluded hypothesis testing), and cross-tabulated frequencies and proportions. We used Chi-square tests for bivariate comparisons of socio-demographic characteristics and health status with three outcomes of interests: vaccination status, likelihood of being vaccinated if offered a $50 gift card, and extent to which incentives influenced trust in COVID-19 vaccination. Missing values were limited and were excluded from adjusted analyses.

© 2023 Faherty LJ et al. *JAMA Network Open.*

Analyses were conducted using Stata MP 17.0 (StataCorp, LLC, College Station, TX). Statistical significance was a 2-sided *P*<.05.

eTable. Survey Questions and Response Options
Note that due to space limitations, results from selected questions are presented in this article.

Question Stem	Response Options
Q1. Have you been vaccinated against COVID-19?	○ Yes, I'm fully vaccinated and boosted<br>○ Yes, I'm fully vaccinated but not boosted<br>○ Yes, I've gotten the first dose of a 2-dose series, so I'm partially vaccinated<br>○ No, but I might get vaccinated in the future<br>○ No, I don't plan to get vaccinated against COVID-19
(if yes to Q1)<br>Q2. Did you receive an incentive for any of your vaccination doses? Examples include a gift card/cash, free food or drink, free haircut, free tickets to a sporting event, paid time off work, being entered into a lottery - even if you didn't win.	○ Yes<br>○ No<br>○ Unsure
(if yes to Q2)<br>Q3. What incentive(s) did you receive?	[free text]
(if yes to Q2)<br>Q4. To what extent did the incentive influence your decision to get vaccinated against COVID-19?	○ The incentive didn't change my decision about getting vaccinated. I would have gotten vaccinated anyway.<br>○ The incentive made me a little more likely to get vaccinated, but I probably would have gotten vaccinated without it.<br>○ The incentive made me much more likely to get vaccinated. I might not have gotten vaccinated without it.<br>○ I wouldn't have gotten vaccinated without an incentive.
(if no to Q1)<br>Q5. If you were offered a $50 gift card as an incentive to get vaccinated against COVID-19, this incentive:	○ Would make me much more likely to get vaccinated.<br>○ Would make me slightly more likely to get vaccinated.<br>○ Would not make a difference – I would not be more or less likely to get vaccinated.<br>○ Would make me slightly less likely to get vaccinated.<br>○ Would make me much less likely to get vaccinated.

© 2023 Faherty LJ et al. *JAMA Network Open.*

Q6. To what extent do incentives influence your trust in COVID-19 vaccination?	○ They make me trust vaccination a lot more.<br>○ They make me trust vaccination a little more.<br>○ They do not affect my trust in vaccination.<br>○ They make me trust vaccination a little less.<br>○ They make me trust vaccination a lot less.

© 2023 Faherty LJ et al. *JAMA Network Open.*
