# Supplementary material for: Perceived Influence of Incentives on COVID-19 Vaccination Decision-making and Trust
Source: JAMA Netw Open. 2023 May 19;6(5):e2313436. doi: 10.1001/jamanetworkopen.2023.13436 (PMC10199349; doi:10.1001/jamanetworkopen.2023.13436)
Supplement: Supplement 2. — Data Sharing Statement [file jamanetwopen-e2313436-s002.pdf]

Data Sharing Statement

Faherty. Perceived Influence of Incentives on COVID-19 Vaccination Decision-making and Trust. *JAMA Netw Open*. Published May 19, 2023. doi:10.1001/jamanetworkopen.2023.13436

Data
Data available: Yes
Data types: Deidentified participant data, Data dictionary
How to access data: Data from every study conducted with the ALP is available for download after a certain embargo period. Registration for approved researchers is free.
https://www.rand.org/research/data/alp/data-access.html
When available: beginning date: 07-18-2023

Supporting Documents
Document types: None

Additional Information
Who can access the data: Researchers who request use of the data and are approved.
Types of analyses: For specified research questions.
Mechanisms of data availability: Through an online application using link above.
Any additional restrictions: This research will be presented as an abstract (oral presentation) at the June 2023 AcademyHealth Annual Research Meeting in Seattle.
